# Supplementary material for: Differential Responses of Medicago truncatula NLA Homologs to Nutrient Deficiency and Arbuscular Mycorrhizal Symbiosis
Source: Plants (Basel). 2023 Dec 11;12(24):4129. doi: 10.3390/plants12244129 (PMC10748377; doi:10.3390/plants12244129)
Supplement: Supplementary file 1 [file plants-12-04129-s001.zip › plants-2726494-supplementary.pdf]

Supplementary Materials

Table S1 The sequence of primers used for plasmid construction.

construct	Primer sequence		The purpose of plasmid construction
pDONR221-MtNLA1/MtNLA2	Forward	GGGGACAAGTTTGTACAAAAAAGCAGGCTTCATGAAGTTCTGCAAAAAATACCAG	The plasmid was used to generate GFP-tagged fusion proteins driven by CaMV 35S promoter via LR reaction.
	Reverse	GGGGACCACTTTGTACAAGAAAGCTGGGTCGACGCCCATGAAAGCTCT	
pDONR221-MtNLA3.1	Forward	GGGGACAAGTTTGTACAAAAAAGCAGGCTTCATGAAGTTCTGCAAGACCTATC	The plasmid was used to generate overexpressing *MtNLA3.1* and GFP-tagged fusion proteins driven by CaMV 35S promoter via LR reaction.
	Reverse	GGGGACCACTTTGTACAAGAAAGCTGGGTCGATGCCCATGAATGCTCTA	
pDONR221-MtNLA3.2	Forward	GGGGACAAGTTTGTACAAAAAAGCAGGCTTCATGTCAGAAATAGTGGGATGC	The plasmid was used to generate overexpressing *MtNLA3.2* and GFP-tagged fusion proteins driven by CaMV 35S promoter via LR reaction.
	Reverse	GGGGACCACTTTGTACAAGAAAGCTGGGTCGATGCCCATGAATGCTCTA	
pDONR221-MtNLA3.3	Forward	GGGGACAAGTTTGTACAAAAAAGCAGGCTTCATGAGAATGGACGTGTGGA	The plasmid was used to generate overexpressing *MtNLA3.3* and GFP-tagged fusion proteins driven by CaMV 35S promoter via LR reaction.
	Reverse	GGGGACCACTTTGTACAAGAAAGCTGGGTCGATGCCCATGAATGCTCTA	
pDL-Nub-MtNLA3.1	Forward	ATACGGATCCATGAAGTTCTGCAAGACC	The plasmid was used to express NubG-MtNLA3.1 fusion protein in yeast cells to test protein-protein interaction via LR reaction.
	Reverse	TACAGAATTCGATGCCCATGAATGCTCTA	
pDL-Nub-MtNLA3.1^{SPX}	Forward	ATACGGATCCATGAAGTTCTGCAAGACC	The plasmid was used to express NubG-MtNLA3.1^{SPX} fusion protein in yeast cells to test protein-protein interaction.
	Reverse	TACAGAATTCCTACAGATCAATATCAATTTTGATGGA	
pDL-Nub-MtNLA3.2	Forward	CGCTGGATCCATGTCAGAAATAGTGGGATGC	The plasmid was used to express NubG-MtNLA3.2 and MtNLA3.2-Nub fusion proteins in yeast cells to test protein-protein interaction.
pDL-MtNLA3.2-Nub	Reverse	TACAGAATTCGATGCCCATGAATGCTCTA	
pDL-Nub-MtNLA3.3	Forward	CGCTGGATCCATGAGAATGGACGTGTGGA	The plasmid was used to express NubG-MtNLA3.3 and MtNLA3.3-Nub fusion protein in yeast cells to test protein-protein interaction.
pDL-MtNLA3.3-Nub	Reverse	TACAGAATTCGATGCCCATGAATGCTCTA	
pDL-MtNLA1/2-Nub	Forward	CGCTGGATCCATGAAGTTCTGCAAAAAATACCAG	The plasmid was used to express NubG-MtNLA1/2 fusion protein in yeast cells to test protein-protein interaction.
	Reverse	TATCGAATTCGACGCCCATGAAAGCTCT	
pDL-MtNLA3.1^{SPX}*-Nub*	Forward	ATACGGATCCATGAAGTTCTGCAAGACC	The plasmid was used to express MtNLA3.1^{SPX} -NubG fusion protein in yeast cells to test protein-protein interaction.
	Reverse	TACAGAATTCCAGATCAATATCAATTTTGATGGA	
pAMBV4-MtPT1	Forward	TCTAGAATGTCTGGAGAATTAGGAGT	The plasmid was used to express MtPT1-Cub fusion protein in yeast cells to test protein-protein interaction.
	Reverse	AGGCCTGAAGCTACATTTTCAAACCG	
pAMBV4-MtPT4	Forward	TCTAGAATGGGATTAGAAGTCCTTGA	The plasmid was used to express MtPT4-Cub fusion protein in yeast cells to test protein-protein interaction.
	Reverse	AGGCCTTGCATCTTCTCAGTTCTTGAGTC	

Table S2 The sequence of primers used for qPCR.

Gene	Accession number	Primer sequence		Reference
MtNLA1/MtNLA2	Medtr7g108840/ Medtr8g058603	Forward	TGCTGTGCATTTGGAAGAAC	
		Reverse	AGACGCCCATGAAAGCTCTA	
MtNLA3	Medtr1g088660	Forward	CCGAGAGGCAGGAGTTTATG	
		Reverse	TGCCCATGAATGCTCTACAC	
MtNLA3-1	Medtr1g088660	Forward	CGTGCGCTGATTCTCTTTCTCTTG	
		Reverse	CTTCTGCTCCTGAGCTTGCAT	
MtNLA3-2	Medtr1g088660	Forward	GGAGGGTGAGTGTGACTCGT	
		Reverse	CAGTTTCTGTGCACGCTGAT	
MtNLA3-3	Medtr1g088660	Forward	TACTGTGGCAAAGGATGCAG	
		Reverse	ACCTCCACACGTCCATTCTC	
MtBCP1	Medtr1g105120	Forward	TCCATGGTTTTGCTTTCCTC	[51]
		Reverse	CGGTGGAAAGTGCTTCATTT	
MtIPD3	Medtr5g026850	Forward	GCGCTCAAGAAAAATGGCTGAAGC	[66]
		Reverse	GCTTTAGTGATCGAACTTCCTTCTCAAGG	
MtNRT2.1	Medtr4g057890	Forward	AAATACAACTCCTACCCACTAG	
		Reverse	CAATATTAACTTATTAAAGCATGGG	
MtPT4	Medtr1g028600	Forward	GACACGAGGCGCTTTCATAGCAGC	[51]
		Reverse	GTCATCGCAGCTGGAACAGCACCG	
MtRAM1	Medtr7g027190	Forward	AAGCCATTTTCGAGGCGTTT	[51]
		Reverse	CGTTAAGCATCGTCCGGTTT	
MtSTR	Medtr8g107450	Forward	TTCCAATGATGCAGTCCCA	[51]
		Reverse	TGGTTATGACTGCAAATGTGAG	
MtVapyrin	Medtr6g027840	Forward	GGAGGTGAGGACAATCCAAA	[68]
		Reverse	GTCCTGATTCAGCAGCACAA	
RiTub	GLOIN_2v1648057	Forward	TGTCCAACCGGTTTTAAAGT	[67]
		Reverse	AAAGCACGTTTGGCGTACAT	
MtEF1-α	Medtr6g021805	Forward	TGACAGGCGATCTGGTAAGG	[51]
		Reverse	TCAGCGAAGGTCTCAACCAC	

Reference:

[51] Park, H.J. et al, Hyphal branching during arbuscule development requires *Reduced Arbuscular Mycorrhiza1*. *Plant Physiol* **2015**, *169*, 2774-2788

[66] Messinese, E. et al. A novel nuclear protein interacts with the symbiotic DMI3 calcium- and calmodulin-dependent protein kinase of *Medicago truncatula*. *Mol Plant Microbe Interact* **2007,** *20*, 912-921

[67] Wang M. et al. Blumenols as shoot markers of root symbiosis with arbuscular mycorrhizal fungi. *Elife* **2018**, *7*, e37093.

[68] Lindsay, P.L. et al. A Phosphate-Dependent Requirement for Transcription Factors IPD3 and IPD3L During Arbuscular Mycorrhizal Symbiosis in *Medicago truncatula*. *Mol Plant Microbe Interact* **2019**, *32*, 1277-1290.

```
                    SPX domain 1
OsNLA1 MKFAKKYEKYMKGMDE--ELPGVGLKRLKKLLKKCRSDLQSHEN--DGSSAGRCPGHCSV 56
AtNLA1 MKFCKKYEEYMQGQKEKKNLPGVGFKKLKKILKRCRRNHVPSRISFTDAINHNCSRECPV 60
MtNLA1 MKFCKKYQEYMQGQEQ-KKLPEVGFKKLKKILKKCRRDSS---------QSQPCPDHCSV 50
MtNLA2 MKFCKKYQEYMQGQEQ-KKLPEVGFKKLKKILKKCRRDSS---------QSQPCPDHCSV 50
MtNLA3 MKFCKTYQEYMQAQEQ-KKLPVVGFKKLKKIMKKCRRSSQ---------FHKPCPDQCPL 50
       ***.*.*::**:. .:  :** **:*:***::*:** .               *  .* :

               SPX domain 2
OsNLA1 CDGSFFPSLLNEMSAVIGCFNEKAKKLLELHLASGFKKYTMWFTSKGH-KSHGALIQQGK 115
AtNLA1 CDGTFFPELLKEMEDVVGWFNEHAQKLLELHLASGFTKCLTWLRGNSRKKDHHGLIQEGK 120
MtNLA1 CDGTFFPSLLSEMSEIVGCFNQRAQKLLELHLASGFRKYILWFKGKYH-KNHTALIQEGE 109
MtNLA2 CDGTFFPSLLSEMSEIVGCFNQRAQKLLELHLASGFRKYILWFKGKYH-KNHTALIQEGE 109
MtNLA3 CDGTFFPSLLNEMSEIVGCFNQRAQKLLERHLASGFQKYILMLKGKSK-RNHSTLIHEGR 109
       ***:***.**.**. ::* **::*:**** ****** *    : .: : :.*  **::*.

               SPX domain 3
OsNLA1 DLVTYAIINAVAMRKILKKYDKIHYSKQGQEFKAQAQSLHIEILQSPWLCELMAFYMNLR 175
AtNLA1 DLVNYALINAVAIRKILKKYDKIHESRQGQAFKTQVQKMRIEILQSPWLCELMAFHINLK 180
MtNLA1 DLVTYALINATAIRKILKKYDKVHYSKQGQLFKSQVQSMHKEILQSPWLCELMAFHINLR 169
MtNLA2 DLVTYALINATAIRKILKKYDKVHYSKQGQLFKSQVQSMHKEILQSPWLCELMAFHINLR 169
MtNLA3 DLVTYALINAVAIRKILKKYDKIHYSKQGQLFKSQAQTMHKEILQSPWLIELMALHINLR 169
       ***.**:***.*:*********:* *:*** **:*.*.:: ******** ****:::**:

                                                       RING domain
OsNLA1 RSKKNNGA---------MELFGDCSLVFDDDKPTISCNLFDSMRVDISLTCSICLDTVFD 226
AtNLA1 ESKKESGATITSPPPPVHALFDGCALTFDDGKPLLSCELSDSVKVDIDLTCSICLDTVFD 240
MtNLA1 ETKVN--------SRKETALFDECSLTFKDGKPSLTCDLFDSIKIDIDLTCSICLDTVFD 221
MtNLA2 ETKVN--------SRKETALFDECSLTFKDGKPSLTCDLFDSIKIDIDLTCSICLDTVFD 221
MtNLA3 ETKDK--------PRKATALFNGCCLTFKDGKPSLACELFDSIKIDIDLTCSICLDTVFD 221
       .:* :              **. *.*.*.*.** ::*:* **:::**.************

                         RING domain
OsNLA1 PVALSCGHIYCYLCSCSAASVTIVDGLKSAERKSKCPLCRQAGVFPNAVHLDELNMLLSY 286
AtNLA1 PISLTCGHIYCYMCACSAASVNVVDGLKTAEATEKCPLCREDGVYKGAVHLDELNILLKR 300
MtNLA1 PVSLTCGHIFCYICACSAASVSIVDGLKAANPKEKCPLCRERAVYEGAVHLEELNILLGR 281
MtNLA2 PVSLTCGHIFCYICACSAASVSIVDGLKAANPKEKCPLCRERAVYEGAVHLEELNILLGR 281
MtNLA3 PVSLTCGHIFCYSCACSAASVTIVDGLKETHSKEKCPMCREAGVYEGAVHLEELNILLGK 281
       *::*:****:** *:******.:***** :. ..***:**: .*: .****:***:**

OsNLA1 SCPEYWEKRIQMERVERVRLAKEHWESQCRAFLGM 321
AtNLA1 SCRDYWEERRKTERAERLQQAKEYWDYQCRSFTGI 335
MtNLA1 SCQEYWEQRLQSERVERIKQIKEHWDSQCRAFMGV 316
MtNLA2 SCQEYWEQRLQSERVERIKQIKEHWDSQCRAFMGV 316
MtNLA3 SCKEYWEERLQMERVERVKQAKEHWETQCRAFMGI 316
       ** :***:* : **.**::  **:*: ***:* *:
```

Figure S1. The amino acid sequence alignment of MtNLAs with AtNLA1 and OsNLA1. Amino acid sequence labeled in bold is bipartite NLS.

SPX domain 1

```
AtNLA        ---------------MKFCKKYEEYMQGQKEKKNLPGVGFKKLKKILKRC 35
MtNLA3.3     MRMDVWRWLEAADGIYSVKAVYKEIMGKE-A------IIYQH-------- 35
MtNLA3.1     ---------------MKFCKTYQEYMQAQ-EQKKLPVVGFKKLKKIMKKC 34
MtNLA3.2     -------------------------------------------------- 0
```

SPX domain 2

```
AtNLA        RRNHVPSRISFTDAINHNCSRECPVCDGTFFPELLKEMEDVVGWFNEHAQ 85
MtNLA3.3     -FIN---------LLFFAQEDVLYVCDGTFFPSLLNEMSEIVGCFNQRAQ 75
MtNLA3.1     RRSS---------QFHKPCPDQCPLCDGTFFPSLLNEMSEIVGCFNQRAQ 75
MtNLA3.2     -------------------------------------MSEIVGCFNQRAQ 13
                                                  *.::** **::**
```

SPX domain 3

```
AtNLA        KLLELHLASGFTKCLTWLRGNSRKKDHHGLIQEGKDLVNYALINAVAIRK 135
MtNLA3.3     KLLERHLASGFQKYILMLKGKSK-RNHSTLIHEGRDLVTYALINAVAIRK 124
MtNLA3.1     KLLERHLASGFQKYILMLKGKSK-RNHSTLIHEGRDLVTYALINAVAIRK 124
MtNLA3.2     KLLERHLASGFQKYILMLKGKSK-RNHSTLIHEGRDLVTYALINAVAIRK 62
             **** ****** * :  *:*:*: ::*  **:**:***.***********
```

SPX domain 3

```
AtNLA        ILKKYDKIHESRQGQAFKTQVQKMRIEILQSPWLCELMAFHINLKESKKE 185
MtNLA3.3     ILKKYDKIHYSKQGQLFKSQAQTMHKEILQSPWLIELMALHINLRETKD- 173
MtNLA3.1     ILKKYDKIHYSKQGQLFKSQAQTMHKEILQSPWLIELMALHINLRETKD- 173
MtNLA3.2     ILKKYDKIHYSKQGQLFKSQAQTMHKEILQSPWLIELMALHINLRETKD- 111
             ********* *:*** **:*.*.*: ******** ****:****:*:*.
```

RING domain

```
AtNLA        SGATITSPPPPVHALFDGCALTFDDGKPLLSCELSDSVKVDIDLTCSICL 235
MtNLA3.3     -------KPRKATALFNGCCLTFKDGKPSLACELFDSIKIDIDLTCSICL 216
MtNLA3.1     -------KPRKATALFNGCCLTFKDGKPSLACELFDSIKIDIDLTCSICL 216
MtNLA3.2     -------KPRKATALFNGCCLTFKDGKPSLACELFDSIKIDIDLTCSICL 154
                     *  . ***:**.***.**** *:*** **:*:**********
```

RING domain

```
AtNLA        DTVFDPISLTCGHIYCYMCACSAASVNVVDGLKTAEATEKCPLCREDGVY 285
MtNLA3.3     DTVFDPVSLTCGHIFCYSCACSAASVTIVDGLKETHSKEKCPMCREAGVY 266
MtNLA3.1     DTVFDPVSLTCGHIFCYSCACSAASVTIVDGLKETHSKEKCPMCREAGVY 266
MtNLA3.2     DTVFDPVSLTCGHIFCYSCACSAASVTIVDGLKETHSKEKCPMCREAGVY 204
             ******:*******:** ********.:***** :.:.****:*** ***
```

```
AtNLA        KGAVHLDELNILLKRSCRDYWEERRKTERAERLQQAKEYWDYQCRSFTGI 335
MtNLA3.3     EGAVHLEELNILLGKSCKEYWEERLQMERVERVKQAKEHWETQCRAFMGI 316
MtNLA3.1     EGAVHLEELNILLGKSCKEYWEERLQMERVERVKQAKEHWETQCRAFMGI 316
MtNLA3.2     EGAVHLEELNILLG-SCKEYWEERLQMERVERVKQAKEHWETQCRAFMGI 253
             :*****:****** **::***** : **.**::****:*: ***:* ***
```

Figure S2. The amino acid sequence alignment of MtNLA3 variants.

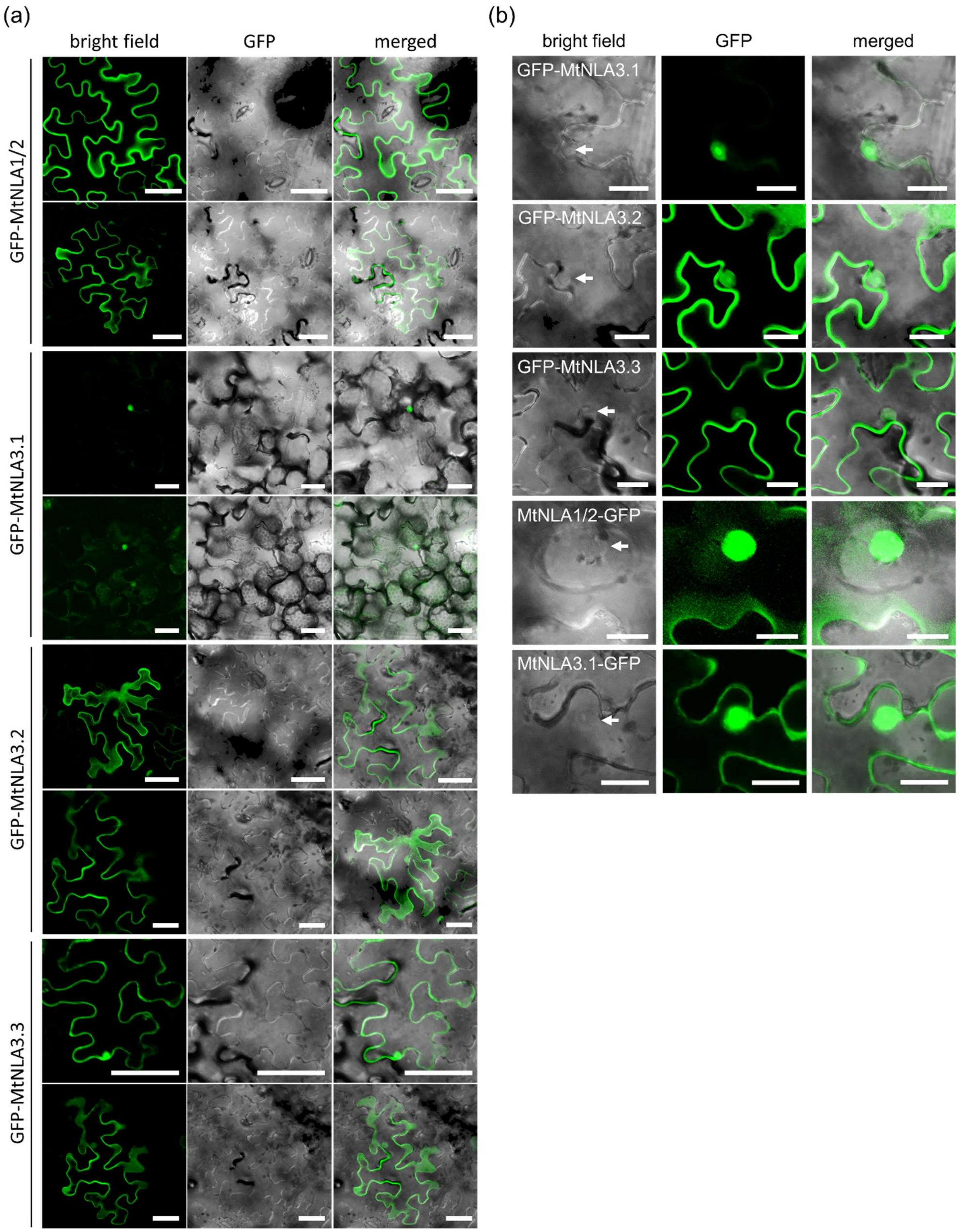

Figure S3. The localization of GFP-tagged MtNLA1 and MtNLA3 variants (a) and the closeup view of GFP-tagged MtNLA1 and MtNLA3 variants in the nucleus The white arrows indicate the nucleus in the epidermal cells. Bars means 50 and 25 µm in (a) and (b), respectively.

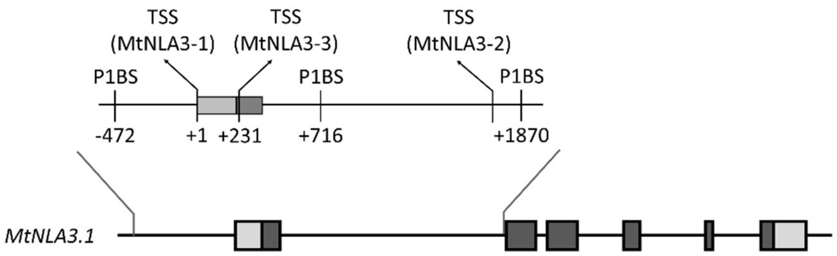

Figure S4. The location of P1BS motifs in MtNLA3 gene. TSS indicates transcriptional start site.

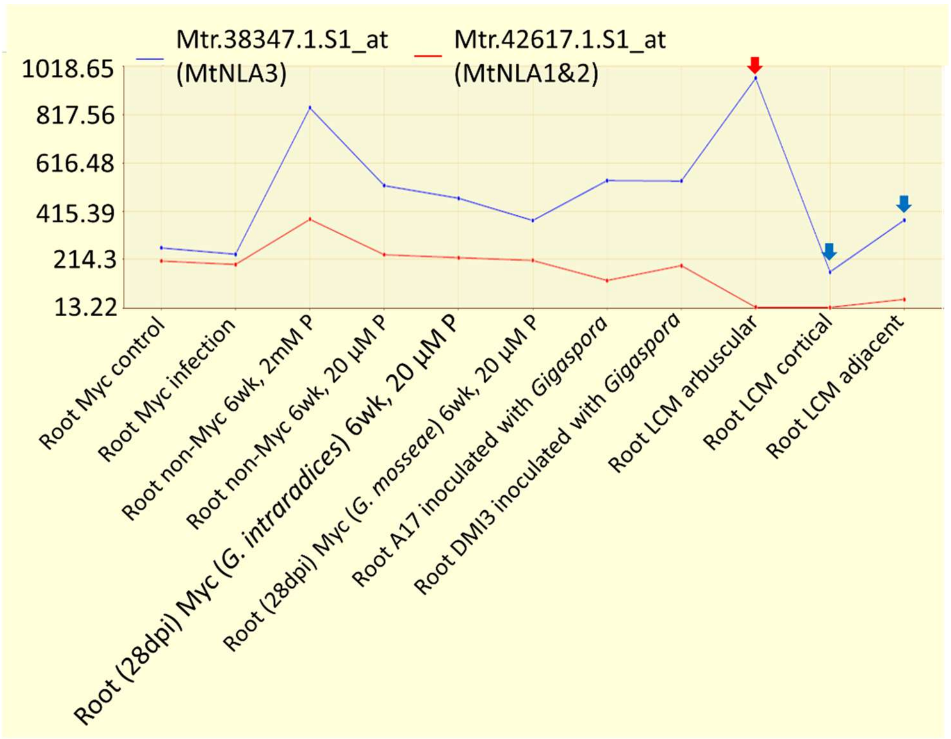

Figure S5. Gene expression profiles of *MtNLA1/MtNLA2* (red line) and *MtNLA3* (blue line) during AM symbiosis. The expression levels of *MtNLA3* are higher in arbuscule-containing cortical cells (red arrow) than in adjacent or mock-treated cortical cells (blue arrows). The profiles are extracted from *Medicago truncatula* Gene Expression Atlas (http://mtgea.noble.org/v2/).
